# Opportunities for Individual- and Population-Specific Adaptations in Food Is Medicine: A Scoping Review

**DOI:** 10.1016/j.advnut.2026.100684

**Published:** 2026-06-18

**Authors:** Caroline Owens, Marcela D Radtke, Juna Hatta-Langedyk, Julia Markmann, Ainsley Fleming-Wood, Kenia Zepeda Carrillo, Laisha Martinez-Reyes, Yiding Zhao, Jennifer Woo Baidal, Lisa G Rosas

**Affiliations:** 1 Department of Anthropology, University of North Carolina Wilmington, Wilmington, NC, United States; 2 Food is Medicine Institute, Friedman School of Nutrition Science and Policy, Tufts University, Boston, MA, United States; 3 Department of Epidemiology and Population Health, Stanford University School of Medicine, Palo Alto, CA, United States; 4 Department of Pediatrics, Stanford University School of Medicine, Stanford, CA, United States; 5 School of Public Health, University of California, Berkeley, CA, United States; 6 Department of Nutritional Sciences and Toxicology, University of California, Berkeley, CA, United States; 7 Department of Medicine, Division of Primary Care and Population Health, Stanford University School of Medicine, Palo Alto, CA, United States

**Keywords:** food is medicine, nutrition interventions, disease states, cultural adaptation, life course

## Abstract

Food is Medicine (FIM) interventions are a promising approach for addressing food insecurity and nutrition-sensitive chronic conditions. Despite emerging literature on FIM, the nature and extent of adaptations within programs have not been systematically evaluated. Therefore, the objective of this scoping review was to synthesize evidence on individual- and population-specific adaptations in FIM interventions, including medically tailored meals (MTM), medically tailored groceries (MTG), and produce prescriptions (PRx). A comprehensive search of PubMed, Web of Science, and Cumulative Index of Nursing and Allied Health Literature was conducted in January 2025 using keywords related to FIM. Study participant and intervention characteristics were summarized, and adaptations were categorized according to: *1*) age and household size; *2*) disease state; or *3*) culture and community. In total, 6266 abstracts were screened, and 89 peer-reviewed manuscripts were included in the review. The FIM interventions included MTM (*n* = 20), MTG (*n* = 18), and PRx (*n* = 51), and were mostly conducted in adult populations (71.9%). Most manuscripts (80.9%) included ≥1 population-specific adaptation, most commonly for disease state (46.1%) or culture and community (43.8%). Nearly all studies on MTM and MTG mentioned adaptations for disease state (95.0% and 66.7%, respectively) compared with a relatively smaller proportion of adaptations among PRx (19.6%). Disease state adaptations most commonly included aligning food provisions with evidence-based dietary guidelines. In contrast, a higher proportion of PRx and MTG mentioned adaptations specific to culture and community (51.0% and 50.0%, respectively) compared with MTM (20.0%). These adaptations varied widely, but included the translation of study materials, community engagement in intervention development, and client-choice models of food provision. FIM interventions are being implemented across the United States, with varying levels of adaptation to address unique needs of target populations. The findings highlight opportunities for further research on strategies to enhance adaptation, improve reach and effectiveness, and ensure the sustainability of FIM programs across diverse communities.

## Introduction

**Statement of Significance**

Improving the characterization of adaptations within Food is Medicine (FIM) interventions is essential to ensure they effectively address age-specific, disease state, and cultural needs of diverse individuals and populations. This scoping review evaluates adaptations used in FIM interventions, highlighting important considerations for their design, implementation, and evaluation.

Nutrition is a central and modifiable determinant of leading causes of morbidity and mortality in the United States and globally [1]. Nutrition insecurity and food insecurity, both social drivers of health, are frequently targeted by United States programs and policies seeking to advance health equity [[2], [3], [4], [5]]. Food is Medicine (FIM) interventions include a range of healthcare-integrated programs, many of which are supported by state-level policies [6], which have proliferated across the United States in the past decade [7,8]. By leveraging the existing infrastructure of the United States healthcare system, FIM interventions aim to improve access to nutritious foods, health outcomes, and health equity [7,8]. Compared with previously implemented dietary and lifestyle interventions, FIM more directly addresses structural barriers to healthy dietary patterns through the provision of clinically prescribed nutritious groceries or meals. FIM interventions range from medically tailored meals (MTM) designed for patients with complex, nutrition-sensitive chronic conditions and high healthcare utilization to broader, preventive approaches, such as medically tailored groceries (MTG) and produce prescriptions (PRx), for individuals at risk for chronic conditions [7].

Each of these approaches can be modified and tailored based on context, target population, and partnerships. MTMs are generally prepared under the supervision of registered dietitian nutritionists (RDNs) to meet the specific nutritional needs of patients living with chronic conditions, such as heart disease, diabetes, HIV, renal failure, and cancer [9]. In this regard, MTM, by definition, are specifically tailored for a given disease state or health condition [10]. However, modifications based on other factors, including patient age or cultural and religious dietary preferences, are likely more variable across FIM interventions. MTG, a less-intensive approach for a broader range of patients, provide patients with cash or debit card–based vouchers or access to healthy groceries, such as vegetables, fruits, grains, beans, lean proteins, and/or dairy, and may be implemented with or without tailoring for disease status or household size [3]. PRx similarly provide patients with fruits and vegetables that can be directly provided in a produce box or through cash or debit card–based vouchers that can be redeemed at local farmers’ markets, grocery stores, or mobile markets, among other locations [3]. As MTG and PRx are often recommended more broadly for disease prevention or lower-risk medical conditions, there may be less individual- or population-specific adaptations included.

A growing body of research, including findings from systematic reviews and meta-analyses, suggests that FIM programs can improve food security status [11,12], fruit and vegetable consumption [[11], [12], [13]], and health outcomes, such as hemoglobin A1c (HbA1c) and body mass index (BMI) [14]. Simulation studies also suggest that FIM interventions may positively impact healthcare utilization and expenditure in the United States, including 1.6 million averted hospitalizations and net cost savings of $13.6 billion annually [6,15]. Notably, however, the implementation of FIM interventions varies widely depending on the population of interest, local context, clinical status, and social needs [16]. There is limited evidence describing how FIM adaptations are made and how they influence program effectiveness, generalizability, and scalability [4]. Adaptations in FIM often align with the eligibility criteria and reimbursement guidelines from funders, such as the United States Centers for Medicare and Medicaid Services through health-related social needs pilot programs, Medicare Advantage plans, the USDA Gus Schumacher Nutrition Incentive Program, and private healthcare systems, among others [17]. These eligibility criteria may inform FIM adaptations for age, disease state, socioeconomic status, food insecurity, language, and culturally valued foodways (e.g., recipes, familial eating practices, or meal preparation), as well as other programmatic components [18]. Although these adaptations may be required for eligibility and funding, the specific details of the FIM study design may not be reported or published in a systematic way. This lack of standardization in documenting FIM adaptations creates challenges for identifying which components are essential versus population or context specific. As a result, a critical gap remains in characterizing adaptations to better understand the impact on health outcomes, improve generalizability, and scale effective models across diverse communities and settings [19].

Despite the rapidly advancing field of FIM, no published literature systematically analyzes how these programs are adapted to meet diverse population needs. Given the emphasis on integrating precision nutrition—defined as an individual’s response to food based on genetic, behavioral, environmental, and lifestyle factors—into FIM [20], there is a need to better understand the range of both individual- and population-specific adaptations in existing interventions. In this scoping review, we address this evidence gap by identifying and categorizing the adaptations implemented for age and household size, disease state, and culture and community described in extant peer-reviewed studies on FIM interventions conducted in the United States and highlight gaps and opportunities for future research and clinical practice.

## Methods

The methods and findings were conducted in accordance with the PRISMA extension for Scoping Review checklist [21]. The protocol for this scoping review was registered on the Open Science Framework [22].

### Literature search strategy

A systematic search was performed in alignment with the Population, Intervention, Comparator, and Outcome framework to identify relevant FIM literature, emphasizing intervention adaptations specific to age, disease state, and culture or community. A comprehensive literature search of PubMed, Web of Science, and the Cumulative Index of Nursing and Allied Health Literature (CINAHL) was performed in January 2025. The primary search terms included FIM-related terminology, including “Produce Prescription Programs,” “Medically Tailored Groceries,” “Medically Tailored Meals,” among other healthcare-integrated food-based interventions (Supplemental Table 1). The articles identified in the comprehensive literature search were uploaded into Covidence for the preliminary title and abstract review.

### Eligibility criteria and screening

The preliminary title and abstract review were performed independently by 2 reviewers (JHL, JM, AFW, KZC, LMR, YZ), with discrepancies mediated by a third reviewer (CO, MDR). The preliminary screening criteria emphasized studies conducted in the United States as healthcare and nutrition practices may differ in other countries. The screening protocol also included food-based interventions that indicated a healthcare-integrated component, ranging from a FIM referral from a healthcare provider to a clinic-based food pantry program. Food-based interventions conducted in a community setting without a healthcare component were excluded. Controlled feeding studies that included specific functional foods were excluded, as the present analysis focused on interventions designed to supplement habitual diets with whole foods. Studies synthesizing data across multiple FIM interventions were excluded, as aggregated data may limit the ability to identify which intervention approach was adapted or tailored. To ensure all relevant literature was captured in the screening process, there were no explicit exclusion criteria pertaining to the type of adaptation reported in the FIM intervention, as specific age, disease, or culture and community are not always detailed in the limited word count of an abstract. After the preliminary title and abstract review, articles that met the initial inclusion criteria were reviewed in full text independently by 2 reviewers (JHL, JM, AFW, KZC, LMR, YZ) and conflicts were resolved through adjudication by a third reviewer (CO, MDR).

### Data extraction

The following data elements were extracted in duplicate by 2 reviewers: study design, target population and demographic characteristics (e.g., location, mean age, race/ethnicity, biological sex or gender, etc. as reported by the authors), the type of FIM intervention, how the FIM intervention was delivered, the duration of the intervention, primary outcomes of interest, and the details on if and how the FIM intervention was adapted or tailored for different age groups, disease status, or culture and community characteristics. Adaptations or tailoring to the FIM interventions were interpreted within the context of the reported methods. Strategies were categorized using modified typologies of cultural adaptation strategies from Kreuter et al. [23], alongside an adapted version of the Resnicow et al. [24] framework for interpreting depth and sensitivity of strategies. Strategy types, operational definitions, and corresponding sensitivity levels are detailed in Table 1 [23,24]. Two authors independently reviewed and classified each adaptation according to this schema (CO, MDR), with discrepancies resolved through consensus discussion.

**TABLE 1:** Description of adaptation strategies used to tailor Food is Medicine interventions

Strategy type	Strategy description^1^	Sensitivity level^2^
Peripheral	Use of supplemental strategies outside of core components of an intervention. To enhance accessibility or effectiveness of an intervention.	Surface
Evidential	Use of data on a given health issue or using data to inform intervention components within the population/community. To enhance the relevance of an intervention component for a given group.	Surface (evidence is applied across populations) Deep (evidence informs personalization or individualization for each participant)
Linguistic	Use of dominant, traditional, or preferred language. To make programs and materials more accessible.	Surface (direct translation of study materials) Deep (translation of study materials with consideration of cultural relevance)
Constituent-involving	Drawing directly on the experience of members of the community. To inform the intervention through active engagement in intervention design, content, or components received.	Surface (participant feedback or engagement informs intervention components) Deep (community members serve as codesigners, advisors, or implementers in the intervention)
Sociocultural	Considers the context of broader social and cultural values or characteristics. The cultural values, beliefs, behaviors, and environment of the participants are recognized, reinforced, and built upon to enhance or inform the intervention.	Surface (component of the intervention is altered based on sociocultural characteristics or context) Deep (component of the intervention is grounded in sociocultural characteristics or context)

1 Adapted from: Kreuter et al. [23].

2 Adapted from: Resnicow et al. [24].

### Data synthesis

The findings from the included articles were consolidated into descriptive tables, with continuous variables reported as mean ± SD and count variables reported as *n* (%) by FIM type. Two authors independently coded the adaptations to identify general themes across studies (CO, MDR). The adaptations and modifications were organized by age, disease state, and culture and community before thematic analysis. As several studies reported adaptations across multiple categories, the frequencies presented are not mutually exclusive.

## Results

**FIGURE 1:**
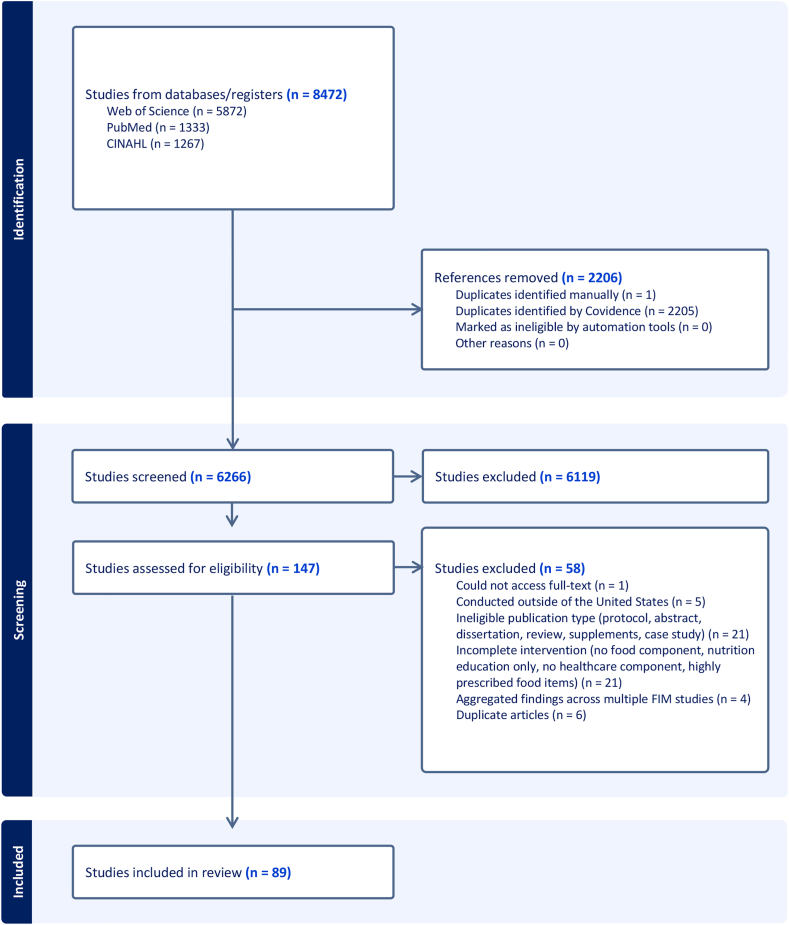
PRISMA diagram detailing inclusion and exclusion criteria. CINAHL, Cumulative Index of Nursing and Allied Health Literature.

The initial database search identified 8472 articles, including 5872 from Web of Science, 1333 from PubMed, and 1267 from CINAHL (Figure 1). A total of 2206 duplicate articles were removed (2205 removed by Covidence, 1 removed manually), and the remaining 6266 studies were screened for eligibility. Studies that did not meet the inclusion criteria were excluded after the initial screening (*n* = 6119), and 147 studies were assessed in full text for eligibility. From these, 58 additional studies were excluded for the following reasons: incomplete FIM intervention (no food component, nutrition education only, no healthcare component: *n* = 21); ineligible publication type (protocol, abstract, dissertation, case report, review: *n* = 21); duplicate articles (*n* = 6); outside of the United States (*n* = 5); aggregated findings across multiple FIM studies (*n* = 4); or could not access full text after multiple author contact attempts (*n* = 1). A total of 89 articles met the inclusion criteria and were included in the final scoping review.

Of the 89 included articles, 20 (22.5%) studies reported on MTM, 18 (20.2%) on MTG, and 51 (57.3%) on PRx interventions (Table 2). The study designs consisted of quasi-experimental (49.4%), randomized controlled trials (16.9%), and observational (7.9%), as well as qualitative and mixed-methods approaches (25.8%). The primary target population included adults (71.9%), followed by children and pediatrics (12.4%), whole households (11.2%), and pregnant or postpartum participants (4.5%). A majority of studies (80.9%) detailed specific adaptations that were included in the FIM intervention design. Of the articles that mentioned adaptations, a majority (56.9%) pertained to specific disease states and culture and community (54.2%), with fewer noted for age or household size (20.8%). Additionally, there were FIM interventions that incorporated adaptations spanning multiple domains, including disease state and culture and community (11.2%), age and household size and culture and community (6.7%), and across all 3 domains (3.4%) (Supplemental Table 2). FIM interventions engaged participants experiencing a range of health conditions, including type 2 diabetes (37.4%), hypertension (16.5%), overweight or obesity (16.5%), cardiovascular disease and heart failure (7.7%), cancer (7.7%), HIV (4.4%), high-risk pregnancy or gestational diabetes (4.4%), and multiple unspecified diet-related conditions (12.1%), among others. The reported duration of FIM interventions varied, ranging between 0 and 12 weeks (29.2%), >12 weeks to 6 months (40.4%), and >6 months (30.3%).

**TABLE 2:** Characteristics of studies included in this scoping review classified by FIM intervention type^1^

	MTM (*N* = 20)	MTG (*N* = 18)	PRx (*N* = 51)	Overall (*N* = 89)
Study design				
Observational	0 (0%)	1 (5.6%)	6 (11.8%)	7 (7.9%)
Qualitative subanalysis	5 (25.0%)	1 (5.6%)	17 (33.3%)	23 (25.8%)
Quasi-experimental	7 (35.0%)	11 (61.1%)	26 (51.0%)	44 (49.4%)
Randomized controlled trial	8 (40.0%)	5 (27.8%)	2 (3.9%)	15 (16.9%)
Target population				
Adults	19 (95.0%)	11 (61.1%)	34 (66.7%)	64 (71.9%)
Children and pediatrics	0 (0%)	2 (11.1%)	9 (17.6%)	11 (12.4%)
Pregnant and postpartum	1 (5.0%)	2 (11.1%)	1 (2.0%)	4 (4.5%)
Whole household	0 (0%)	3 (16.7%)	7 (13.7%)	10 (11.2%)
Duration				
0–12 wk	8 (40.0%)	4 (22.2%)	14 (27.5%)	26 (29.2%)
>12 wk to 6 mo	8 (40.0%)	6 (33.3%)	21 (41.2%)	35 (39.3%)
>6 mo	4 (20.0%)	8 (44.4%)	16 (31.4%)	28 (31.5%)
Adaptation				
Age and household size	1 (5.0%)	5 (27.8%)	9 (17.6%)	15 (16.9%)
Culture and community	4 (20.0%)	9 (50.0%)	26 (51.0%)	39 (43.8%)
Disease state	19 (95.0%)	12 (66.7%)	10 (19.6%)	41 (46.1%)
Adaptations not detailed	0 (0%)	2 (11.1%)	15 (29.4%)	17 (19.1%)

Abbreviations: FIM, Food is Medicine; MTG, medically tailored groceries; MTM, medically tailored meals; PRx, produce prescription programs.

1 Counts and percentages may not sum to column totals, as studies may have detailed multiple adaptation types.

### Age and household size

**FIGURE 2:**
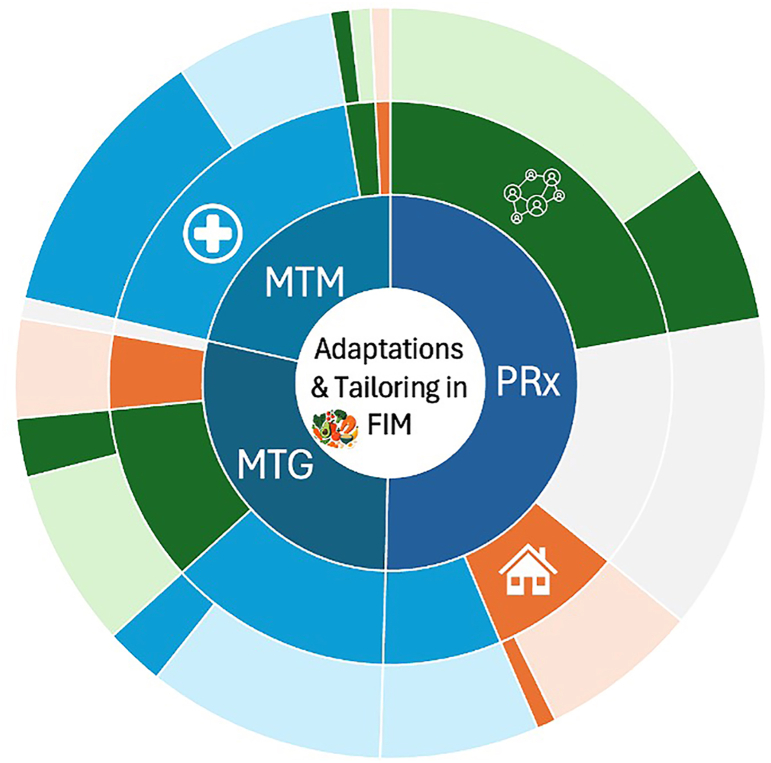
Hierarchical breakdown of adaptations and tailoring in FIM interventions included in this scoping review, organized by intervention type, adaptation category, and degree of adaptation. Segment size is proportional to the number of interventions, with color differentiation at the second level indicating adaptation or tailoring type (blue represents disease state, orane represents age and household size, and green represents culture and community). Color saturation distinguishes tailoring level (surface in lighter saturation vs. deep in darker saturation). Disease state culture and community age and household size. Adaptations not detailed. FIM, Food is Medicine; MTG, medically tailored groceries; MTM, medically tailored meals; Rx, produce prescription.

Reflecting the small number of studies focused on pediatric or older adult populations, few studies explicitly mentioned adaptations specifically focused on certain age groups or scaled to household size (*n* = 15; 16.9%) (Figure 2; Table 3 [[25], [26], [27], [28], [29], [30], [31], [32], [33], [34], [35], [36], [37], [38], [39]]). Among studies that did target specific age groups, adaptations were most evident in pediatric populations. Age-specific adaptations included using health-related educational curricula, such as the *Healthy Habits, Happy Homes* [34] or the Family Lifestyle Program’s Produce Prescription Initiative (FliPRx 2.0) [40] to target younger audiences. Some age-related adaptations overlapped with broader adjustments to include a whole household approach, including tailoring for household size [[26], [27], [28], [29],^35^,^38^,^39^]. Household size was accounted for in many pediatric and whole household studies, where the amount of food provisions was scaled to the number of people per household (e.g., 12 meals per household member up to a family unit of 5 [38,39]) or the dollar amount was increased (e.g., $15 for a family of 3, $20 for a family of 4, $25 for a family of ≥5 [27] or $1 per family member per day [28,29]). Most of the studies detailing age and household-size adaptations were surface level, drawing on the sociodemographic characteristics of participating communities to inform core components of the intervention. Of the 15 studies employing these strategies, one used a deep strategy by incorporating a curriculum designed specifically for children [34], and one drew on constituent perspectives to inform voucher denomination [31].

**TABLE 3:** Adaptations in Food is Medicine for age and household size (*n* = 15)

Author and year	Study objective	Study design; FIM type	Target population and sample size	FIM duration	Adaptation type	Adaptation overview	Adaptation strategy
Arnold et al., 2024 [25]	To examine changes in produce consumption, food security, and anthropometrics over a 24-mo period.	Quasi-experimental; PRx	Whole household; *n* = 55 households	>6 mo	Age and household size	Food boxes were tailored for household size.	Surface; sociocultural
Biber, 2023 [26]	To determine the effectiveness of the FIM program that was implemented at a regional hospital among participants with diabetes.	Quasi-experimental; MTG	Adults; *n* = 20	>6 mo	Age and household size	Groceries were tailored for household size, such that foods were enough to feed each patient’s entire family, twice a day, for the 2-wk period.	Surface; sociocultural
Burrington et al., 2020 [27]	To evaluate if a fruit and vegetable PRx, cooking/nutrition classes and an online produce market would improve dietary intake and family meal patterns during and after the intervention.	Quasi-experimental; PRx	Whole household; *n* = 10 parents, *n* = 29 children	>12 wk to 6 mo	Age and household size	Tailored for household size ($15 for a family of 3, $20 for 4, and $25 for 5 or more was given to each family).	Surface; sociocultural
Cook et al., 2021 [28]	To evaluate improvements in food security and health outcomes associated with participation in a PRx.	Quasi-experimental; PRx	Whole household; *n* = 159	>12 wk to 6 mo	Age and household size	Voucher amount was adjusted for household size ($1 per household member per day).	Surface; sociocultural
Cook et al., 2023 [29]	To assess the relationship between PRx attendance and changes in cardiovascular risk factors.	Quasi-experimental; PRx	Whole household; *n* = 331	>12 wk to 6 mo	Age and household size	Voucher amount was adjusted for household size ($1 per household member per day).	Surface; sociocultural
Esquivel et al., 2020 [30]	To explore feasibility of a community-based pediatric PRx and explore the facilitators and barriers to participation.	Quasi-experimental; PRx	Children and pediatrics; *n* = 193	0–12 wk	Age and household size	Parents received a PRx for each eligible child.	Surface; sociocultural
Esquivel et al., 2022 [31]	To understand motivators, barriers, and support for PRx participation in a population of Native Hawaiian and Other Pacific Islanders.	Quasi-experimental (qualitative subanalysis); PRx	Children and pediatrics; *n* = 25 parents, *n* = 34 children	>12 wk to 6 mo	Age and household size	Due to low participation [30], the voucher amount increased from $24 to $50 to account for household size.	Surface; constituent-involving, sociocultural
Ferris et al., 2025 [32]	To examine the impact of an integrated fresh food provision and counseling program during pregnancy.	Quasi-experimental; MTG	Pregnant/postpartum; *n* = 125	>6 mo	Age and household size	Food shares were scaled to household size.	Surface; sociocultural
Hu et al., 2022 [33]	To assess the effect of a hospital-based food pantry clinic on self-reported dietary changes, health outcomes, and resource utilization.	Quasi-experimental; MTG	Whole household; *n* = 79	>6 mo	Age and household size	A volunteer shopping assistant ensured appropriate quantities of food based on household size.	Surface; sociocultural
Jones et al., 2020 [34]	To assess the impact of a PRx on the health outcomes and behaviors of participating children.	Quasi-experimental; PRx	Children and pediatrics; *n* = 243	>12 wk to 6 mo	Age and household size	The *Healthy Habits, Happy Homes* childhood obesity curriculum was used.	Deep; peripheral, sociocultural
Lyonnais et al., 2022 [35]	To examine the impact of a PRx on fruit and vegetable intake and examine the effectiveness of partnering with local retailers in implementing a PRx.	Quasi-experimental (qualitative subanalysis); PRx	Adults; *n* = 22 interviews, *n* = 93 survey	>6 mo	Age and household size	Additional vouchers of $5 were distributed to households with children.	Surface; sociocultural
Newman et al., 2021 [36]	To examine the facilitators and barriers affecting positive outcomes of a PRx from the perspective of program providers.	Qualitative subanalysis; PRx	Adults; *n* = 15	>12 wk to 6 mo	Age and household size	Voucher amount was adjusted for household size ($1 per household member per day).	Surface; sociocultural
Rivera et al., 2023 [37]	To determine the feasibility of a dietitian-led lifestyle intervention to address food access, nutrition literacy, cooking skills, and hypertension among safety-net primary care adult patients.	Quasi-experimental; MTM^1^	Adults; *n* = 13	>12 wk to 6 mo	Age and household size	Additional servings for meals and meal kits were provided based on the number of household members who routinely eat meals with the index participant.	Surface; sociocultural
Woo Baidal et al., 2022 [38]	To examine reach, feasibility, and retention of Food FARMacia, a clinically based food insecurity intervention, among children aged <6 y.	Observational; MTG	Children and pediatrics; *n* = 50	>12 wk to 6 mo	Age and household size	The food amounted to ∼12 meals per household member for ≤5 household members twice monthly.	Surface; sociocultural
Woo Baidal et al., 2023 [39]	To test whether children in Food FARMacia, a clinically based food insecurity intervention, have smaller age-adjusted, sex-specific BMIz gains than matched counterparts.	Quasi-experimental; MTG	Children and pediatrics; *n* = 265	>12 wk to 6 mo	Age and household size	The food amounted to ∼12 meals per household member for ≤5 household members twice monthly.	Surface; sociocultural

Abbreviations: FIM, Food is Medicine; MTG, medically tailored groceries; MTM, medically tailored meals; PRx, produce prescription programs.

1 This intervention was primarily MTM, but participants were able to select meal kits and/or grocery boxes which can also be classified as MTG.

### Disease state

In studies that documented adaptations based on disease state, the involvement of RDNs, dietetic technicians, or other nutrition professionals was consulted to guide dietary recommendations and nutrition support tailored to individual disease states (*n* = 24) (Table 4 [26,32,33,37,[41], [42], [43], [44], [45], [46], [47], [48], [49], [50], [51], [52], [53], [54], [55], [56], [57], [58], [59], [60], [61], [62], [63], [64], [65], [66], [67], [68], [69], [70], [71], [72], [73], [74], [75], [76], [77]]). Many studies, particularly those reporting on MTMs, noted that meals were developed in accordance with dietary guidelines established by professional organizations, including the American Diabetes Association [41,59,60,62,63,68,76], American Heart Association [41,59,60,63,48], and Academy of Nutrition and Dietetics [46,57]. Others were informed by evidence-based dietary patterns such as the Dietary Approaches to Stop Hypertension (DASH) diet [55,66,67,69,70,72,75,78], the Mediterranean diet [37,53], MyPlate [47], and accreditation standards established by the FIM Coalition [52]. Most adaptations for disease state were informed by evidence-based dietary guidelines, with the majority classified as deep-level evidential strategies. Of the 41 studies detailing these adaptations, 26 incorporated evidential strategies at a deep level, reflecting more individualized or professional-guided tailoring to participants’ medical and nutrition-related needs. Surface-level evidential strategies were more common in PRx and MTG interventions, where food provision was aligned with established dietary guidelines but lacked individual-level tailoring. A smaller subset of studies integrated constituent-involving strategies, and 3 incorporated sociocultural strategies that contextualized health conditions within relevant cultural contexts or provided culturally-informed MTMs [51,60,72].

**TABLE 4:** Adaptations in Food is Medicine for disease state (*n* = 41)

Author and year	Study objective	Study design; FIM type	Target population and sample size	FIM duration	Adaptation type	Adaptation overview	Adaptation strategy
Belak et al., 2022 [41]	To evaluate the impact of home-delivered, MTM, and MNT among food-insecure patients following hospitalization for congestive heart failure compared with retrospectively matched controls.	Quasi-experimental; MTM	Adults; *n* = 156	>12 wk to 6 mo	Disease state	Meals followed the American Diabetes Association guidelines for carbohydrate intake and balanced protein and fat macronutrients, and sodium content was aligned with American Heart Association guidelines. MNT emphasized patients’ adherence to evidence-based dietary guidelines for congestive heart failure.	Deep; constituent-involving, evidential
Berkowitz et al., 2018 [42]	To determine whether either home-delivered MTM or nontailored food resources reduces healthcare utilization and costs among dually Medicare and Medicaid eligible adults.	Quasi-experimental; MTM	Adults; *n* = 3077	>6 mo	Disease state	A registered dietitian tailored the MTM to the participant’s medical needs across 17 dietary tracks.	Deep; evidential
Berkowitz et al., 2018 [43]	To test whether a MTM delivery program improved dietary quality in individuals with type 2 diabetes and food insecurity.	Randomized controlled trial; MTM	Adults; *n* = 44	0–12 wk	Disease state	A registered dietitian tailored the MTM to be suitable for diabetes and the participant’s personal medical needs across 17 dietary tracks.	Deep; evidential
Berkowitz et al., 2019 [44]	To determine whether participation in a MTM intervention is associated with fewer hospitalizations.	Quasi-experimental; MTM	Adults; *n* = 1020	>6 mo	Disease state	A registered dietitian tailored the meals to the participant’s medical needs.	Deep; evidential
Berkowitz et al., 2020 [45]	To understand the experiences of MTM program participants.	Randomized controlled trial (qualitative subanalysis); MTM	Adults; *n* = 20	>12 wk to 6 mo	Disease state	A registered dietitian tailored the MTM to be suitable for diabetes and the participant’s personal medical needs across 17 dietary tracks.	Deep; evidential
Biber et al., 2023 [26]	To determine the effectiveness of a MTG program for adults with type 2 diabetes.	Quasi-experimental; MTG	Adults; *n* = 20	>6 mo	Disease state	A registered nurse provided diabetes education and an individualized care plan. A certified health and wellness coach provided group and individual sessions for disease management.	Deep; constituent-involving, evidential
Boxer et al., 2022 [46]	To determine if 2 vs. 4 wk of MTM after hospitalization improves patient outcomes.	Randomized controlled trial; MTM	Adults; *n* = 650	0–12 wk	Disease state	Meals adhered to standards established by Academy of Nutrition and Dietetics, providing 4 dietary choices all appropriate for patients living with diabetes. A registered dietitian assisted with meal selection.	Deep; constituent-involving, evidential
Cheyne et al., 2020 [47]	To assess the effectiveness of a food bank–delivered intervention aimed at improving food security and reducing risk factors for type 2 diabetes.	Quasi-experimental; MTG	Adults; *n* = 244	>6 mo	Disease state	Groceries were aligned with the USDA MyPlate and Choose Healthy Options Program, for example, only canned products low in sodium and added sugars were provided to participants.	Surface; evidential
Clark et al., 2024 [48]	To examine the impact of MTM and MNT on clinical outcomes among adults with type 2 diabetes.	Randomized controlled trial; MTM	Adults; *n* = 74	0–12 wk	Disease state	Meals were tailored for type 2 diabetes based on the American Heart Association diet. MNT was provided by a registered dietitian and included diabetes-friendly dietary approaches.	Deep; constituent-involving, evidential
Doyle et al., 2023 [49]	To test whether an intensive FIM program for patients with diabetes and food insecurity improves glycemic control and affects health care use.	Randomized controlled trial; MTG	Adults; *n* = 500	>6 mo	Disease state	Groceries were curated by a dietitian and nurses provided preventive care, such as foot examinations.	Surface; peripheral
Ferris et al., 2025 [32]	To examine the impact of an integrated fresh food provision and counseling program during pregnancy.	Quasi-experimental; MTG	Pregnant/postpartum; *n* = 125	>6 mo	Disease state	Meals included foods that met nutritional needs during pregnancy. Recipes were created by a chef and registered dietitian to address the nutritional needs of a pregnant person. Participants received increased case management for nutrition support during pregnancy.	Deep; constituent-involving, evidential
Gany et al., 2022 [50]	To examine the impact of food insecurity interventions on cancer outcomes.	Randomized controlled trial; MTG	Adults; *n* = 117	>12 wk to 6 mo	Disease state	Nutritionist input informed tailoring of pantry bags for patients with cancer (whole grains, low sodium, and low glycemic index) for one randomization arm.	Surface; evidential
García-Pérez et al. 2024 [51]	To evaluate the impact of a food intervention program on clinical outcomes, healthcare utilization, food insecurity, and patient satisfaction among individuals living with diabetes and food insecurity.	Quasi-experimental; MTG	Adults; *n* = 46	>6 mo	Disease state	Food boxes included disease-appropriate foods and recipes tailored to the nutritional needs of patients with diabetes. Boxes were combined with active specialty diabetic and cardiovascular care management classes. Participants were able to select from 4 culturally and/or disease-appropriate food boxes (Traditional American, Hispanic, Somali, Heart Disease).	Deep; constituent-involving, evidential, sociocultural
Go et al., 2022 [52]	To evaluate whether MTM improve outcomes in recently discharged adults with nutrition-sensitive conditions compared with usual care.	Randomized controlled trial; MTM	Adults; *n* = 1977	0–12 wk	Disease state	Meals aligned with nutritional recommendations based on Food is Medicine Coalition standards that were supported by national guidelines and consistent with Kaiser Permanente nutritional guidelines tailored prioritizing standards for heart failure, then diabetes, and chronic kidney disease, if present. MTM generally followed the DASH diet. Participants randomized to nutrition counseling were offered sessions provided by a registered dietitian.	Deep; constituent-involving, evidential
Harvey et al., 2023 [53]	To identify barriers and facilitators to adhering to an 8-wk Mediterranean diet intervention among patients undergoing chemotherapy.	Randomized controlled trial (qualitative subanalysis); MTM	Adults; *n* = 29	0–12 wk	Disease state	Participants received education resources focused on a Mediterranean diet, consistent with dietary guidelines and nutritional needs for cancer survivors and participated in a session with a nutrition scientist.	Surface; constituent-involving, evidential
Hu et al., 2022 [33]	To assess the effect of a hospital-based food pantry clinic on self-reported dietary changes, health outcomes, and resource utilization.	Quasi-experimental; MTG	Whole household; *n* = 79	>6 mo	Disease state	A trained dietetic technician assisted patients in determining medically tailored food choices.	Deep; constituent-involving, evidential
Huang et al., 2024 [54]	To evaluate the feasibility and acceptability of home-delivered MTM in pregnant patients with diabetes.	Quasi-experimental (qualitative subanalysis); MTM	Pregnant/postpartum; *n* = 24	>12 wk to 6 mo	Disease state	Diabetes-specific MTM were designed using evidence-based dietary guidelines. Registered Dietitians reviewed and approved participants for either 3 or 6 mo of diabetes-specific MTM delivery.	Deep; evidential
Joshi et al., 2018 [55]	To describe implementation of a PRx program for participants with hypertension.	Quasi-experimental (qualitative subanalysis); PRx	Adults; *n* = 231 (*n* = 224 participants; *n* = 7 providers)	0–12 wk	Disease state	Providers adapted nutrition education handouts to align with the DASH diet.	Surface; evidential
Kalarchian et al., 2016 [56]	To evaluate the utility of a structured dietary intervention to assist bariatric surgery patients with weight management.	Randomized controlled trial; MTM	Adults; *n* = 40	>12 wk to 6 mo	Disease state	Participants assigned to the intervention received portion-controlled foods (Nutrisystem), as well as a personalized meal plan that aligned with postbariatric surgery dietary guidelines.	Surface; evidential
Kelly et al., 2023 [57]	To measure the impact of a MTM intervention on participants’ self-reported recovery and satisfaction while recovering from a recent hospitalization.	Qualitative subanalysis; MTM	Adults; *n* = 551 (*n* = 22 interviews; *n* = 529 surveys)	0–12 wk	Disease state	Meals aligned with specific dietary standards established by the Academy of Nutrition and Dietetics for patients with chronic conditions.	Surface; evidential
Kerver et al., 2023 [58]	To assess the feasibility of implementing a food-based intervention to increase fiber intake among pregnant women in a rural setting.	Randomized controlled trial; MTG	Pregnant/postpartum; *n* = 27	0–12 wk	Disease state	Grocery content was primarily designed by registered dietitians to align with dietary guidelines for pregnancy.	Surface; evidential
Palar et al., 2017 [59]	To evaluate the feasibility, acceptability, and potential impact of a medically appropriate food assistance intervention among people living with HIV and/or type 2 diabetes.	Quasi-experimental; MTM	Adults; *n* = 72	>12 wk to 6 mo	Disease state	Carbohydrate and saturated fat levels were set based on recommendations from the American Diabetes Association and American Heart Association, respectively. Nutritionists informed intervention design.	Surface; evidential
Palar et al., 2025 [60]	To investigate the benefits of medically tailored food programs for people with HIV.	Randomized controlled trial; MTM	Adults; *n* = 191	>12 wk to 6 mo	Disease state	MTM were consistent with guidelines for patients with HIV and informed by carbohydrate guidelines from the American Diabetes Association and saturated fat and sodium guidelines from the American Heart Association, with individual tailoring for calories and specialized dietary preferences. Registered dietitians informed intervention design and led nutrition counseling sessions that addressed HIV and other topics.	Deep; constituent-involving, evidential, sociocultural
Rivera et al., 2023 [37]	To determine the feasibility of a dietitian-led lifestyle intervention to concurrently address food access, nutrition literacy, cooking skills, and hypertension among safety-net primary care adult patients.	Quasi-experimental; MTM^1^	Adults; *n* = 13	>12 wk to 6 mo	Disease state	Clinical dietitians led intervention design. Meals contained <500 mg of sodium, and most had <15 g of sugar and <60 g of total carbohydrates per serving.	Surface; evidential
Rothpletz-Puglia et al., 2024 [61]	To assess participants’ perceptions and experiences while participating in a MTM plus intensive nutrition counseling intervention.	Qualitative subanalysis; MTM	Adults; *n* = 20	>6 mo	Disease state	MTM were nutritionally tailored to meet the needs of cancer patients. Registered dietitians on the study prescribed weekly MTM based upon nutrition impact symptoms from 11 different types of meals.	Deep; constituent-involving, evidential
Sastre et al., 2023 [62]	To evaluate the use, barriers, and impact of a delivery-based PRx.	Quasi-experimental; PRx	Adults; *n* = 40	>12 wk to 6 mo	Disease state	Intervention resources were based on recommendations from the American Diabetes Association.	Surface; evidential
Sastre et al., 2023 [63]	To examine the impact of a PRx on food literacy, lifestyle behaviors, and health outcomes of rural, uninsured patients with type2 diabetes.	Quasi-experimental; PRx	Adults; *n* = 56	>12 wk to 6 mo	Disease state	Intervention resources aligned with recommendations from the American Diabetes Association, the American Heart Association, and the USDA.	Surface; evidential
Sastre et al., 2023 [64]	To examine the development, implementation, acceptability, and impact of a PRx with tailored culinary-focused nutrition education.	Quasi-experimental; PRx	Adults; *n* = 54	0–12 wk	Disease state	Nutrition education (recipes and cooking demonstrations) focused on nonstarchy vegetables and were tailored to be low in salt, saturated fat, and starch/sugar for cardiometabolic comorbidities.	Surface; evidential
Sautter et al., 2024 [65]	To describe the sociodemographic and health profile of clients participating in a MTM program and estimate change in health outcomes.	Quasi-experimental; MTM	Adults; *n* = 1959	>12 wk to 6 mo	Disease state	Registered dietitians reviewed and approved participants for either 3 or 6 mo of MTM delivery based on disease-severity. Meal modifications were available for individual needs (kidney-friendly, diabetic/heart healthy, low lactose, low fiber, mild spice, mechanical soft, pureed, and high calorie and protein).	Deep; evidential
Schlosser et al., 2019 [66]	To better understand how participants experience a PRx for adults with hypertension.	Qualitative subanalysis; PRx	Adults; *n* = 30	0–12 wk	Disease state	Intervention resources were adapted to align with the DASH diet. Participants were educated on how to read nutrition facts labels to control salt intake. Each study visit included tailored dietary counseling to improve blood pressure control.	Surface; constituent-involving, evidential
Schlosser et al., 2019 [67]	To examine how economic constraint influence participant experience in a PRx.	Qualitative subanalysis; PRx	Adults; *n* = 23	0–12 wk	Disease state	Intervention resources were adapted to align with the DASH diet. Participants were educated on how to read nutrition facts labels to control salt intake. Each study visit included tailored dietary counseling to improve blood pressure control.	Surface; constituent-involving, evidential
Short et al., 2023 [68]	To assess the feasibility of a food-based diabetes self-management education and support intervention for people with type 2 diabetes and food insecurity.	Quasi-experimental (qualitative subanalysis); MTG	Adults; *n* = 21	0–12 wk	Disease state	Food packages were aligned with the American Diabetes Association nutrition therapy recommendations for high-fiber and low-refined carbohydrate foods. Participants received two 30-min visits with a registered dietitian trained in the intervention. Participants also received DSMES materials from the American Diabetes Association.	Deep; constituent-involving, evidential
Slagel et al., 2022 [69]	To examine impacts of a PRx on participants’ and nonparticipants’ experiences and perceptions of farm direct settings.	Quasi-experimental; PRx	Adults; *n* = 46	>12 wk to 6 mo	Disease state	PRx aligned with DASH dietary recommendations.	Surface; evidential
Slagel et al., 2023 [70]	To examine changes in participants’ fruit and vegetable consumption, nutrition-related knowledge and behavior, and food purchasing practices.	Quasi-experimental; PRx	Adults; *n* = 54	>12 wk to 6 mo	Disease state	PRx aligned with DASH dietary recommendations.	Surface; evidential
Stroud et al., 2023 [71]	To examine the impact of a pilot PRx with tailored education and culinary resources for rural patients with type 2 diabetes in under resourced communities on behavioral and clinical outcomes.	Quasi-experimental; PRx	Adults; *n* = 40	>12 wk to 6 mo	Disease state	Nutrition education and healthy lifestyle handouts focused on diabetes management and PRx aligned with nutrition guidelines for diabetes.	Surface; evidential
Taniguchi et al., 2024 [72]	To evaluate acceptability and feasibility of a culturally tailored food box intervention for improving health outcomes among Chickasaw Nation adults with uncontrolled hypertension.	Randomized controlled trial; MTG	Whole household; *n* = 262	>12 wk to 6 mo	Disease state	Groceries were selected to align with the DASH diet. Intervention development was part of a larger community-based participatory research project (Natives-Controlling Hypertension and Risk Through Technology) with tailoring to improve blood pressure and cardiovascular disease risk factors among native communities.	Deep; constituent-involving, evidential, sociocultural
Tapper et al., 2020 [73]	To determine the feasibility and impact of a low-sodium MTM intervention among patients with cirrhosis and ascites.	Randomized controlled trial; MTM	Adults; *n* = 40	0–12 wk	Disease state	Meals were developed by a registered dietitian to meet the needs of patients with ascites. Intervention resources on nutrition education focused on low-sodium diets.	Surface; evidential
Toma et al., 2024 [74]	To assess the impact of a food prescription program on food security among patients in an underserved medical setting.	Quasi-experimental; MTG	Adults; *n* = 409	>12 wk to 6 mo	Disease state	PhD nutritionists and clinic physicians collaborated with Feeding San Diego to provide guidance for tailoring groceries for those with diabetes and hypertension.	Deep; evidential
Trapl et al., 2018 [75]	To evaluate intervention effectiveness on patient usage of farmers markets and fruit and vegetable consumption.	Quasi-experimental (qualitative subanalysis); PRx	Adults; *n* = 224	0–12 wk	Disease state	Providers adapted nutrition education handouts to align with the DASH diet.	Surface; evidential
Williams et al., 2022 [76]	To test whether a cooking intervention with food provision and diabetes self-management education and support improves HbA1c and diabetes management among adults with type 1 or type 2 diabetes.	Randomized controlled trial; MTG	Adults; *n* = 48	0–12 wk	Disease state	Intervention resources were developed and taught by a registered dietitian certified in diabetes care and included educational support in alignment with the American Diabetes Association.	Surface; evidential
Yu et al., 2022 [77]	To evaluate whether participation in a MTM program improved food security and health outcomes among people living with HIV and food insecurity.	Quasi-experimental; MTM^1^	Adults; *n* = 191	>6 mo	Disease state	A registered dietitian provided nutritional counseling and support as needed.	Surface; constituent-involving

Abbreviations: DASH, Dietary Approaches to Stop Hypertension; DSMES, Diabetes Self-Management Education and Support; FIM, Food is Medicine; HbA1c, hemoglobin A1c, MNT, medical nutrition therapy; MTG, medically tailored groceries; MTM, medically tailored meals; PRx, produce prescription programs.

1 This intervention was primarily MTM, but participants were able to select meal kits and/or grocery boxes which can also be classified as MTG.

### Culture and community

Culture and community adaptations were designed to address a range of sociocultural factors, such as the inclusion of linguistically and culturally-informed intervention materials, as well as strategies to support social drivers of health [79], including intervention components to assist with food insecurity and transportation limitations in diverse communities (Table 5 [12,25,[31], [32], [33], [34],[36], [37], [38], [39],^51^,^62^,^64^,^65^,[69], [70], [71], [72],^74^,^77^,^78^,[80], [81], [82], [83], [84], [85], [86], [87], [88], [89], [90], [91], [92], [93], [94], [95], [96]]). Language adaptations encompassed handouts in the target population’s language and availability of translators. Cultural adaptations included supporting local food systems and providers. Community-engaged intervention codevelopment strategies were included in multiple studies, which involved community members offering direct input on FIM program components, such as culturally relevant foods or recipes and educational materials [32,34,40,72,84,86,[93], [94], [95], [96]]. Some studies used a customer- or client-choice model to allow participants to self-select food items and food quantities based on household preferences [25,38,39]. Sociodemographic limitations were also considered, with adaptations like increased dollar amount for food vouchers given to those with food insecurity (e.g., participants with food insecurity received $20 in redeemable vouchers, whereas participants without food insecurity received $10 [80]) and tailored resources, such as budget-friendly recipes or lower-cost food items to address low-income status [62,64,69,70,78,71,74]. Adaptations for differing literacy levels, such as limiting education materials to elementary or high school-aged reading levels, were also included for varying levels of education attainment [62,64,71]. Additionally, 2 studies provided transportation vouchers to increase FIM intervention engagement and adherence [33,97]. The inclusion of RDNs and other nutritional professionals was also incorporated into studies to enhance adaptations and increase nutrition education more broadly, such as through providing grocery store tours, teaching participants how to read food labels, and recipe development, among other cultural and community-centered practices [36,85,88,90,92,96]. Most adaptations within the culture and community category were surface level, commonly integrating sociocultural and constituent-involving strategies to align food provision and educational resources within the cultural backgrounds, preferences, and lived experiences of participants. Nine of the studies used deep-level strategies, predominantly through constituent-involving approaches such as community advisory boards or participant-informed designs. Linguistic strategies including direct translation of intervention materials and more culturally grounded adaptations were also evident. Several studies incorporated multiple strategy types, highlighting the intersection of sociocultural, linguistic, and constituent-involving tailoring in FIM interventions.

**TABLE 5:** Adaptations in Food is Medicine for culture and community (*n* = 39)

Author and year	Study objective	Study design; FIM type	Target population and sample size	FIM duration	Adaptation type	Adaptation overview	Adaptation strategy
Abel et al., 2022 [80]	To assess whether PRx redemption was associated with food insecurity, sociodemographic characteristics, and health measures	Observational; PRx	Children and pediatrics; *n* = 242	>12 wk to 6 mo	Culture and community	Participants who screened positive for food insecurity received a $20 PRx, while those who screened as food secure received a $10 PRx. Spanish-speaking patients were contacted using a certified interpreter service.	Surface; linguistic, sociocultural
Arnold et al., 2024 [25]	To examine changes in produce consumption, food security, and anthropometrics.	Quasi-experimental; PRx	Whole Household; *n* = 55 households	>6 mo	Culture and community	A “client choice” PRx model was used to provide participants the ability to choose the type and number of items received in their boxes based on household preferences.	Deep; constituent-involving
Ayala et al., 2023 [81]	To describe and address the facilitators and barriers of PRx implementation and practice from the perspective of clinical staff.	Quasi-experimental (Qualitative subanalysis); PRx	Adults; *n* = 15	>12 wk to 6 mo	Culture and community	Clinical staff provided participants with information about wraparound clinic and community resources, including culturally tailored nutrition education, and on-site produce in addition to the vouchers/EBT.	Surface; sociocultural
Bryce et al., 2021 [82]	To evaluate the effects of a fruit and vegetable prescription program on changes in HbA1c, blood pressure, and BMI in patients with diabetes.	Randomized controlled trial; PRx	Adults; *n* = 112	>12 wk to 6 mo	Culture and community	Principles of community-based participatory research were implemented, such as a CAB to best evaluate the program and research process.	Deep; constituent- involving
Chao et al., 2025 [83]	To examine feasibility and preliminary effects of PRx.	Randomized controlled trial; PRx	Adults; *n* = 32	0–12 wk	Culture and community	Behavioral intervention was tailored for individuals with food insecurity, such as ways to address financial and environmental barriers.	Surface; sociocultural
Esquivel et al., 2022 [31]	To understand factors that participants perceived as motivators, barriers, and support for PRx participation among a population of Native Hawaiian and Other Pacific Islanders.	Quasi-experimental (qualitative subanalysis); PRx	Children and pediatrics; *n* = 59	>12 wk to 6 mo	Culture and community	In response to findings from a feasibility study, the PRx program was adapted for longer duration (3-mo to 6-mo), voucher amount was increased ($24–$50). Vouchers could be redeemed at local farmers market to purchase culturally relevant produce.	Deep; constituent-involving, evidential
Ferris et al., 2025 [32]	To study the impact of an integrated fresh food provision and counseling program during pregnancy.	Quasi-experimental; MTM	Pregnant/postpartum; *n* = 125	>6 mo	Culture and community	The intervention was designed using community-engaged input (Participant Advisory Council) to tailor for culturally relevant foods.	Deep; constituent-involving, sociocultural
Fischer et al., 2022 [84]	To evaluate the feasibility and explore the impact of a PRx program on food insecurity and fruit and vegetable intake in families with young children.	Quasi-experimental; PRx	Whole household; *n* = 25 households	>6 mo	Culture and community	The intervention included ∼24 hours of culturally tailored virtual nutrition education based on SNAP-Ed, delivered through cooking classes, video tips, recipe demonstrations, and instructional cards. The curriculum was culturally adapted to reflect the norms, values, and lived experiences of the local African American community, featuring racially concordant educators and peer-shared recipes and strategies.	Deep; constituent-involving, evidential, sociocultural
García-Pérez et al. 2024 [51]	To evaluate the impact of a food intervention program on clinical outcomes, healthcare utilization, food insecurity, and patient satisfaction among individuals living with diabetes and food insecurity.	Quasi-experimental; MTG	Adults; *n* = 46	>6 mo	Culture and Community	Nutrition and recipe materials were provided in the patient’s preferred language (English, Spanish, or Somali). Participants were able to select from 4 culturally and/or disease-appropriate food boxes (Traditional American, Hispanic, Somali, Heart Disease).	Deep; constituent-involving, evidential, linguistic, sociocultural
Hager et al., 2023 [85]	To evaluate the impacts of a PRx on glycemic control for patients with diabetes.	Quasi-experimental; PRx	Adults; *n* = 786	>12 wk to 6 mo	Culture and community	A registered dietitian-led group-based grocery store lessons.	Surface; peripheral
Hu et al., 2022 [33]	To assess the effect of a hospital-based food pantry clinic on self-reported dietary changes, health outcomes, and resource utilization.	Quasi-experimental; MTG	Whole household; *n* = 79	>6 mo	Culture and community	The clinic provided transportation vouchers to support the grocery pick-up.	Surface; peripheral
Imuro et al., 2023 [86]	To evaluate the impact of a PRx for predominantly Hispanic/Latino adults living with or at risk of type 2 diabetes.	Quasi-experimental; PRx	Adults; *n* = 303	0–12 wk	Culture and community	The study provided outreach materials, communications, and questionnaires in both Spanish and English. Additional questionnaires were collected to assess the impact of PRx on vegetable, tortilla, and soda consumption, which are commonly consumed foods in Hispanic/Latino community	Surface; linguistic, sociocultural
Izumi et al., 2020 [87]	To determine the preliminary effectiveness of CSA Partnerships for Health in improving dietary behaviors, self-efficacy to eat vegetables, food security, and overall health among safety-net clinic patients.	Quasi-experimental; PRx	Adults; *n* = 48	>12 wk to 6 mo	Culture and community	The study provided both Spanish and English communication and used locally sourced produce from a CSA partnership.	Surface; linguistic
Jones et al., 2020 [34]	To assess the impact of a PRx on the health outcomes and behaviors of participating children.	Quasi-experimental; PRx	Children and pediatrics; *n* = 243	>12 wk to 6 mo	Culture and community	Participants were encouraged to purchase healthy traditional Navajo foods with vouchers. The educational sessions were adapted to Navajo context by involving children in sessions and goal setting. Community input informed the design and implementation of the intervention.	Deep; constituent-involving, linguistic, sociocultural
Kleckner et al., 2022 [88]	To evaluate the feasibility of delivering an 8-wk MedDiet intervention during chemotherapy treatment for cancer and the preliminary efficacy of the MedDiet intervention on cancer-related fatigue.	Randomized controlled trial; MTM	Adults; *n* = 33	0–12 wk	Culture and community	Participants completed a one-on-one educational session with a nutrition scientist to discuss strategies for behavior change, including goal setting, self-monitoring, and stimulus control	Surface; constituent-involving
Lumpkin et al., 2023 [89]	To evaluate the impact of a payer-sponsored food delivery and health coaching program on health outcomes, food insecurity, and healthcare cost among Medicaid low-income members with diabetes and food insecurity.	Quasi-experimental; MTG	Adults; *n* = 882	>12 wk to 6 mo	Culture and community	The intervention included weekly communication between participants and a board-certified health and wellness coach to provide more individualized support. Groceries were aligned with the USDA Healthy Plate recommendations.	Surface; constituent-involving, evidential
Muleta et al., 2024 [40]	To evaluate the impact of a pediatric PRx on food security and nutrition-related behaviors	Quasi-experimental; PRx	Children and pediatrics; *n* = 82 families	>12 wk to 6 mo	Culture and community	The intervention included ∼24 hours of virtual nutrition education based on SNAP-Ed, delivered through cooking classes, video tips, recipe demonstrations, and instructional cards, tailored to reflect the norms, beliefs, and lived experiences of the predominantly African American local community. The program also aimed to involve racially concordant educators. Families shared recipes, recommendations, and cooking strategies among peers, aligned with the curriculum.	Deep; constituent-involving, evidential, sociocultural
Newman et al., 2021 [36]	To examine the facilitators and barriers affecting positive outcomes of a PRx from the perspective of program providers.	Qualitative subanalysis; PRx	Adults; *n* = 15	>12 wk to 6 mo	Culture and community	Cooking classes were led by bilingual peer educator and cooking classes were led by dietitian nutritionist	Surface; linguistic
Owens et al., 2023 [90]	To address gaps in the current literature to enhance the sustainability, scalability, and transferability of PRx interventions to other hospital and clinical settings.	Quasi-experimental; PRx	Adults; *n* = 863	>6 mo	Culture and community	Patients were invited to meet with a registered dietitian.	Surface; peripheral
Raber et al., 2024 [91]	To understand multiple aspects of implementing a FIM program from the perspective of clinic staff.	Observational; PRx	Adults; *n* = 40	0–12 wk	Culture and community	Intervention was grounded in cultural competency and classes were offered in multiple languages.	Deep; linguistic, sociocultural
Rivera et al., 2023 [37]	To determine the feasibility of a dietitian-led lifestyle intervention to concurrently address food access, nutrition literacy, cooking skills, and hypertension among safety-net primary care adult patients.	Quasi-experimental; MTM^1^	Adults; *n* = 13	>12 wk to 6 mo	Culture and community	Meals and recipes used culturally appropriate and familiar foods.	Surface; sociocultural
Rosas et al., 2025 [12]	To examine the effectiveness of a PRx, Recipe4Health, on health behaviors, food insecurity, mental health, indicators of nutrition-related chronic conditions, prescriptions for medications, and healthcare utilization.	Quasi-experimental; PRx	Adults; *n* = 5286	>12 wk to 6 mo	Culture and community	The intervention incorporated a local community-based organization, who hosted group medical visits facilitated by bilingual/bicultural health coaches.	Surface; constituent-involving, linguistic
Sastre et al., 2023 [62]	To evaluate the use, barriers, and impact of a delivery-based PRx.	Quasi-experimental; PRx	Adults; *n* = 40	>12 wk to 6 mo	Culture and community	Resources were tailored for people with low socioeconomic status, low literacy levels, and included regional food preferences.	Surface; sociocultural
Sastre et al., 2023 [64]	To examine the development, implementation, acceptability, and impact of a PRx with tailored culinary-focused nutrition education.	Quasi-experimental; PRx	Adults; *n* = 54	0–12 wk	Culture and community	Resources were tailored for people with low socioeconomic status, low literacy levels, and included regional food preferences.	Surface; sociocultural
Sautter et al., 2024 [65]	To describe the sociodemographic and health profile of clients participating in a MTM program and estimate change in health outcomes over program participation.	Quasi-experimental; MTM	Adults; *n* = 1959	>12 wk to 6 mo	Culture and community	Patients could select meals that aligned with cultural or religious preferences, including no pork, no beef, and no seafood.	Surface; constituent-involving, sociocultural
Slagel et al., 2022 [69]	To examine impacts of a PRx on participants’ and nonparticipants’ experiences and perceptions of farm direct settings.	Quasi-experimental; PRx	Adults; *n* = 46	>12 wk to 6 mo	Culture and community	Intervention curriculum was adapted for low-income Georgian families, including culturally relevant food.	Surface; sociocultural
Slagel et al., 2023 [70]	To examine changes in participants’ fruit and vegetable consumption, nutrition-related knowledge and behavior, and food purchasing practices.	Quasi-experimental; PRx	Adults; *n* = 54	>12 wk to 6 mo	Culture and community	Intervention curriculum was adapted for low-income Georgian families, including culturally relevant food.	Surface; sociocultural
Slagel et al., 2022 [78]	To examine whether a multilevel PRx intervention model with intensive education improves dietary behaviors, food security, and health outcomes over single-level interventions alone.	Quasi-experimental; PRx	Adults; *n* = 60	>12 wk to 6 mo	Culture and community	Participants were provided 2 SNAP-Ed direct education programs in both English and Spanish: Food Talk and Food Talk: Better U. The Food Talk curriculum was adapted for low-income Georgian families, including culturally relevant food.	Surface; evidential, linguistic, sociocultural
Sneed et al., 2024 [92]	To assess the feasibility and accessibility of a whole foods diet for adults with prediabetes and their offspring.	Quasi-experimental; MTG	Whole household; *n* = 12 children, *n* = 8 caregivers	0–12 wk	Culture and community	A registered dietitian developed menus using the Nutrition Data System for Research. Foods aligned with the food groups presented in the 2020–2025 Dietary Guidelines for Americans. Adults received counseling from the study dietitian.	Surface; constituent-involving, evidential
Stroud et al., 2023 [71]	To examine the impact of a pilot PRx with tailored education and culinary resources for rural patients with type 2 diabetes in under resourced communities on behavioral and clinical outcomes.	Quasi-experimental; PRx	Adults; *n* = 40	>12 wk to 6 mo	Culture and community	Intervention resources were adapted to include images to replace and/or reduce the text to address literacy barriers. Recipes were adapted to be culturally appropriate and were tailored for patients with lower income by focusing on using minimal equipment, inexpensive ingredients, and quick preparation techniques.	Surface; linguistic, sociocultural, peripheral
Suh et al., 2024 [93]	To explore experiences with a PRx among people living with food insecurity and low income in a predominantly African American community.	Qualitative subanalysis; PRx	Adults; *n* = 24	>6 mo	Culture and community	Intervention resources were developed with participant feedback and included culturally relevant recipes and nutrition education.	Deep; constituent-involving, sociocultural
Taniguchi et al., 2024 [72]	To evaluate acceptability and feasibility of a culturally tailored food box intervention for improving health outcomes among Chickasaw Nation adults with uncontrolled hypertension.	Randomized controlled trial; MTG	Whole household; *n* = 262	>12 wk to 6 mo	Culture and community	Intervention development was part of a larger community-based participatory research project (Natives-Controlling Hypertension and Risk Through Technology) with tailoring to improve blood pressure and cardiovascular disease risk factors among Native communities.	Deep; constituent-involving, sociocultural
Toma et al., 2024 [74]	To assess the impact of a food prescription program on food security among patients in an underserved medical setting.	Quasi-experimental; MTG	Adults; *n* = 409	>12 wk to 6 mo	Culture and community	Groceries were adapted for traditional Hispanic recipes based on food distribution. Cooking and nutrition education classes included cultural and economic tailoring.	Surface; sociocultural
Woo Baidal et al., 2022 [38]	To examine reach, feasibility, and retention in Food FARMacia, a clinically based food insecurity intervention among children aged <6 y.	Observational; MTG	Children and pediatrics; *n* = 50	>12 wk to 6 mo	Culture and community	A customer-choice model was implemented to align with the USDA MyPlate guidelines.	Surface; constituent-involving, evidential
Woo Baidal et al., 2023 [39]	To test whether children in Food FARMacia, a clinically based food insecurity intervention, would have smaller age-adjusted, sex-specific BMIz gains than matched counterparts.	Quasi-experimental; MTG	Children and pediatrics; *n* = 265	>12 wk to 6 mo	Culture and community	A customer-choice model was implemented to align with the USDA MyPlate guidelines.	Surface; constituent-involving, evidential
Ylitalo et al., 2023 [94]	To describe patient and physician engagement with a PRx program and to assess changes in cooking self-efficacy, diet-related self-management, and dietary outcomes.	Quasi-experimental; PRx	Adults; *n* = 33	0–12 wk	Culture and community	Recipes were culturally relevant (representing Caribbean, West African, and other cuisines), and nutrition education included substitutions for traditional, culturally specific foods.	Surface; sociocultural
York et al., 2020 [95]	To assess the feasibility of using medical prescriptions of organic vegetables to improve health outcomes among Latinos with type 2 diabetes.	Quasi-experimental; PRx	Adults; *n* = 23	0–12 wk	Culture and community	Vegetable selections were based on a consumer survey of local Latino adults, prioritizing those most preferred and most often eaten.	Surface; constituent-involving, sociocultural
Yu et al., 2022 [77]	To evaluate whether participation in a MTM program improved food security and health outcomes among people living with HIV and food insecurity.	Quasi-experimental; MTM^1^	Adults; *n* = 191	>6 mo	Culture and community	Participants could choose and switch between meals and meal kits. Participants who expressed additional need could receive an additional monthly pantry box.	Surface; constituent-involving, sociocultural
Zimmer et al., 2022 [96]	To elicit the perspectives of individuals with food insecurity who were enrolled in a PRx delivery program through a collaboration between an academic medical center and multiple community partners.	Qualitative subanalysis; PRx	Adults; *n* = 155 (*n* = 18 interviews)	>12 wk to 6 mo	Culture and community	The intervention was developed after gaining community input and materials, including newsletters and recipes were developed by a nutritionist	Deep; constituent- involving

Abbreviations: CAB, Community Advisory Board; CSA, Community-Supported Agriculture; EBT, electronic benefit transfer; FIM, Food is Medicine; HbA1c, hemoglobin A1c; MTG, medically tailored groceries; MTM, medically tailored meals; PRx, produce prescription programs; SNAP-Ed, Supplemental Nutrition Assistance Program Education.

1 This intervention was primarily MTM, but participants were able to select meal kits and/or grocery boxes which can also be classified as MTG.

### Adaptations not detailed

Seventeen of the included studies did not explicitly detail adaptations related to age or household size, disease state, or culture and community considerations in their methodological descriptions (Table 6 [13,[98], [99], [100], [101], [102], [103], [104], [105], [106], [107], [108], [109], [110], [111], [112], [113]]). The limited reporting or absence of adaptations may reflect contextual and methodological constraints, including the use of food banks or food pantries [98,106] or serving large populations [108,110,113], which may limit the capacity to implement individualized adaptations. Additionally, several qualitative and mixed-methods studies provided limited details on FIM study design, as the intervention was not the primary outcome of interest [[99], [100], [101],^104^,^107^,^109^,^112^].

**TABLE 6:** Adaptations not detailed in Food is Medicine interventions (*n* = 17)

Author and year	Study objective	Study design; FIM type	Target population and sample size	FIM duration	Adaptation type	Adaptation overview
Gany et al., 2015 [98]	To examine uptake of an emergency food system at 5 cancer clinics in New York City, hospital-based food pantries, and predictors of use, among low-income urban patients with cancer.	Quasi-experimental; MTG	Adults; *n* = 351	>6 mo	Adaptations not detailed	
Johnson et al., 2023 [99]	To explore pediatric clinicians’ experiences with a PRx, focusing on its impact on their knowledge, attitudes, and practices related to addressing food insecurity in an urban primary care setting.	Quasi-experimental (qualitative subanalysis); PRx	Children and pediatrics; (clinicians: *n* = 11)	0–12 wk	Adaptations not detailed	
Joseph et al., 2023 [100]	To assess the PRx on physical and mental health, and to reflect on the lessons from implementing in a rural area.	Quasi-experimental; PRx	Adults; *n* = 33	0–12 wk	Adaptations not detailed	
Li et al., 2024 [101]	To understand how program participants perceived a card-based pediatric PRx and how it impacted dietary outcomes and food security status.	Qualitative subanalysis; PRx	Whole household; *n* = 23 caregivers	>6 mo	Adaptations not detailed	
Lo et al., 2024 [102]	To assess if participation in a North Carolina PRx for SNAP participants with diet-sensitive health conditions (SuperSNAP) is associated with changes in purchase composition and spending source.	Quasi-experimental; PRx	Adults; *n* = 1440 SuperSNAP, *n* = 45,851 SNAP only	>6 mo	Adaptations not detailed	
Lowrey et al., 2024 [103]	To assess the operational effectiveness and scalability of partnership models of care.	Quasi-experimental; MTG	Adults; *n* = 19,221 food-insecure controls, *n* = 3709 referred to PRx	>6 mo	Adaptations not detailed	
Lyonnais et al., 2022 [104]	To examine voucher redemption rates, change in fruit and vegetable intake, and suggestions for improvement among participants enrolled in a PRx occurring in existing public health programs throughout rural eastern North Carolina.	Quasi-experimental (Qualitative subanalysis); PRx	Adults; *n* = 125 surveys, *n* = 32 interviews	>6 mo	Adaptations not detailed	
Mayfield et al., 2024 [105]	To describe a PRx program for Medicaid-insured patients.	Quasi-experimental; PRx	Adults; *n* = 711	>12 wk to 6 mo	Adaptations not detailed	
Orsega-Smith et al., 2019 [106]	To evaluate a PRx offered to low-income families by local pediatricians and the Food Bank of Delaware.	Quasi-experimental; PRx	Whole household; *n* = 41 households	>6 mo	Adaptations not detailed	
Rhodes et al., 2024 [107]	To describe client experiences and satisfaction with PRx, with an emphasis on the extent to which they felt they were treated with respect and dignity, and identify recommendations for improving client experiences.	Observational (qualitative subanalysis); PRx	Adults; *n* = 17	>6 mo	Adaptations not detailed	
Ridberg et al., 2025 [108]	To examine redemption rates in the PRx program, how outcomes changed over time with expansion of specific program components and duration, and whether individual-level characteristics related to varying trends in outcomes over time.	Observational; PRx	Adults; *n* = 2680	>6 mo	Adaptations not detailed	
Saxe-Custack et al., 2022 [109]	To explore caregiver experiences with access to and utilization of the PRx during COVID-19, and understand perceived changes in the food environment during the “stay home, stay safe” executive order.	Qualitative subanalysis; PRx	Children and pediatrics; *n* = 56	>6 mo	Adaptations not detailed	
Saxe-Custack et al., 2024 [110]	To assess whether exposure to a PRx was associated with differences in child diet, food security, physical activity, weight status, and blood pressure.	Observational; PRx	Children and pediatrics; *n* = 680 dyads	>6 mo	Adaptations not detailed	
Suarez et al., 2024 [111]	To evaluate the congruence between food insecurity screening outcome and clinic-based food pantry utilization and to examine caregiver-reported comfort, motivation, and benefits of utilization.	Quasi-experimental (qualitative subanalysis); PRx	Children and pediatrics; *n* = 120 children, *n* = 14 interviews with caregivers	>6 mo	Adaptations not detailed	
Trapl et al., 2016 [112]	To determine the applicability of integrating a PRx with an existing health care and farmers’ market systems.	Quasi-experimental (qualitative subanalysis); PRx	Pregnant and postpartum; *n* = 75 surveys, *n* = 10 interviews with providers	0–12 wk	Adaptations not detailed	
Xie et al., 2021 [13]	To evaluate a PRx utilization and its effects on healthy food purchasing and diabetes control among participants with type 2 diabetes.	Observational; PRx	Adults; *n* = 699	>6 mo	Adaptations not detailed	
Ylitalo et al., 2024 [113]	To describe a dynamic, multiyear, multilevel process evaluation of an ongoing, cross-sector PRx collaboration among a community health center, a nonprofit farm, and an academic institution.	Observational; PRx	Adults; *n* = 8046	>6 mo	Adaptations not detailed	

Abbreviations: MTG, medically tailored groceries; PRx, produce prescription programs; SNAP, Supplemental Nutrition Assistance Program.

## Discussion

This scoping review identified a range of adaptations across different target populations and FIM programs, including MTM, MTG, and PRx. Of the identified adaptations, those addressing disease state were most common, particularly within MTM interventions. Other strategies included tailoring interventions to household-level and culture and community factors, including household size, culturally valued foodways, socioeconomic status, and experiences of food insecurity, with many studies integrating multiple approaches. Despite the number of unique adaptations observed, this review highlights several gaps in current FIM practices, most notably, limited tailoring for certain populations, such as non-English-speaking communities, children, pregnant individuals, and whole households, involving parents and children as index participants. These findings underscore opportunities to expand FIM interventions to better meet the complex needs of populations currently underserved by existing models.

The depth of adaptations varied across FIM program types. In general, MTM programs incorporated greater individual-level tailoring, including considerations for clinical and dietary needs, along with more counseling over time and longer-term follow-up, likely reflecting the greater medical acuity of the populations served. MTG interventions tended to focus on providing condition-specific foods with educational support, and PRx programs aimed to primarily address access and affordability without a strong depth of tailoring for specific health conditions. Despite the emphasis on disease state adaptations across all FIM programs, with greater emphasis on disease state in MTM and MTG, other forms of adaptation were less common. It should be noted that not all MTGs were tailored for disease state, with many containing healthy groceries reflecting general dietary recommendations for the United States population. Culture and community adaptations were more prominent in PRx and MTG interventions (51.0% and 50.0%, respectively) compared with MTM (20.0%), often limited to the literal translation of materials into another language, primarily Spanish, and age-specific adaptations, most common in MTG (27.8%), rarely extended beyond scaling food for entire households, leaving pediatric-focused approaches largely unexplored.

For the FIM interventions that included adaptations for specific disease states, many of these interventions followed established national dietary guidelines (e.g., American Diabetes Association, American Heart Association)*,* with adaptations supported by the expertise of RDNs. The inclusion of RDNs supports the integration of disease-specific meal composition and clinical recommendations into FIM programs, with their expertise furthering information and nutrition education materials for achieving disease-specific nutrient parameters. Of the studies that tailored for disease state, 64.9% mentioned engaging RDNs or other nutrition professionals. Involving RDNs throughout FIM interventions may enhance their effectiveness, as their specialized training allows them to provide personalized, evidence-based nutrition care for those requiring or benefitting from medically tailored nutrition [9]. Additionally, building on RDNs’ expertise and culturally grounded approaches, personalized nutrition may offer a framework to further customize some FIM interventions by incorporating participants’ biological data such as clinical biomarkers of disease status and their lived experiences, thereby increasing relevance and potential impact [114]. Advances in precision nutrition as part of nutrition counseling and education services may also offer a viable pathway to meet individual- and population-specific dietary needs [20].

In contrast to the extensive adaptations for disease state, adaptations related to age- and household size, as well as culture and community, were reported less frequently or with comparatively limited details. Some FIM programs extended their impact by scaling meals to household size and providing nutrition education for both children and adults in the home. This depth of tailoring reflects the importance of engaging the broader family system in dietary change rather than just the enrolled index patient [115]. Other FIM programs included the provision of culturally relevant foods, recipes that incorporated novel foods into culturally familiar dishes, or translated intervention materials into additional languages, primarily Spanish. Evidence from meta-analyses suggests that culturally adapted behavioral and mental health interventions may be more effective at changing health-related practices than nonadapted interventions [116]. Similarly, culturally adapted nutrition interventions have demonstrated promising outcomes for Indigenous and ethnic minority groups in the United States [117]; however, further research on the types and degree of adaptation, particularly in nutrition-related interventions, is needed [118]. Few studies included community members and target populations in the design of the FIM intervention, known as community codesign [31,32,72,82,93,96]. Community engagement and codesign are viable mechanisms to not only promote health equity but also improve cultural tailoring to participants’ lived experiences and local environments [117,119]. Prioritizing community-engaged approaches may increase the cultural relevance of FIM and simultaneously promote higher engagement and adherence across nutrition interventions.

As FIM interventions are expanded to encompass populations across the life course, identifying age-appropriate ancillary supports, including nutrition education materials, is warranted [120]. Although nutrition education is a core component of FIM programming, only 2 studies described the inclusion of a child-focused curriculum, in which children were the designated recipient of the *Healthy Habits, Happy Homes* [34] and the FliPRx 2.0 curricula [40]. *Healthy Habits, Happy Homes* also included children and their caregivers in the design of the curriculum, which led to child engagement in sessions and goal setting, as well as the use of Diné foods and language within activities implemented through the Navajo Fruit and Vegetable Prescription Program [34]. Given that modifiable chronic disease risk factors, such as diet and lifestyle, are established early in life, incorporating pediatric-focused strategies within FIM interventions may support the development of health-promoting behaviors and reduce the risk of developing chronic diseases later in life.

In addition to adapting FIM interventions for individual- and population-specific factors, it is also important to consider complementary policies, such as federal food assistance, which can help support the sustainability of nutrition services as part of preventive healthcare. At present, many FIM programs are piloted or reimbursed through Medicaid Section 1115 waivers or in lieu of services policies within managed care organizations [17]. On the basis of findings presented in this review, gaps in reach to vulnerable populations receiving public health insurance are likely, particularly among marginalized or historically underserved communities. Concurrently, proposed changes to Medicaid and federal food assistance coverage, including tightened eligibility, may exacerbate nutrition-related disparities and make tailoring efforts less of a priority given limited funding [121]. These proposed policy changes may additionally pose a threat to the expansion of tailored approaches in FIM given its existing reimbursement structures and integration into healthcare systems. In this context, leveraging local community resources may be essential to ensure that FIM interventions remain responsive to the diverse needs of vulnerable populations while operating within existing policy constraints.

### Limitations

The findings in this scoping review should be interpreted within the context of several limitations. First, this review was limited by including only peer-reviewed studies conducted in the United States, which may have excluded findings from gray literature, program evaluations, and reports published by organizations engaged in FIM, as well as FIM interventions conducted in other countries. Due to the overall aims of our study to descriptively synthesize the adaptations used in FIM, a meta-analysis of the effect size of these interventions was not conducted. Such analysis could assess whether programs that involved adaptations were associated with greater overall effectiveness in study outcomes. Finally, this scoping review included all eligible FIM studies regardless of whether adaptations were reported; however, the characterization of adaptations was limited to those explicitly described in the published methods sections. As a result, studies that may have included adaptations but did not detail them in the published methods may have been overlooked. For this reason, it is possible that this scoping review underestimated the number of studies that included adaptations and underrepresented the magnitude or depth of adaptations that may have been included without sufficient detail.

In conclusion, the adaptations synthesized in this scoping review demonstrate the depth and breadth of FIM interventions across diverse populations. The findings highlight that FIM interventions include adaptations at both individual and population levels, emphasizing a lifespan perspective, such as the inclusion of pediatric populations, the integration of RDNs to align meals and food offerings with disease-specific nutritional recommendations, and the incorporation of culture and community-centered adaptations, including expanding language capabilities, food preferences, and community codesign to enhance program reach and effectiveness. This review underscores a need for further research that evaluates tailored FIM interventions on patient health outcomes, implementing comparative effectiveness approaches to determine whether and to what extent tailoring confers added benefits over standard, nontailored FIM interventions across diverse populations and chronic conditions. These findings suggest important next steps, including comprehensive assessment of the impact and cost of adapting FIM to meet the unique needs of specific populations and communities.

## Author contributions

The authors’ responsibilities were as follows – LGR, CO, MDR: conceptualized the study, designed the scoping review protocol; CO, MDR, JH-L, JM, AFW, KZC, LM-R, YZ: performed the screening and data extraction; CO, MDR: conducted data analysis; CO, MDR, JH-L, JM, AFW: drafted the first version of the manuscript; LGR, JWB: provided study oversight; and all authors: provided detailed review and approved the final manuscript.

## Data availability

The data supporting the findings in this scoping review (e.g., data extraction forms, EndNote files, etc.) will be made available from the corresponding author (LGR) on request.

## Declaration of Generative AI and AI-assisted Technologies in the Writing Process

The authors declare that no generative AI or AI-assisted technologies were used in the writing of this manuscript.

## Funding

The authors reported no funding received for this study.

## Conflict of interest

The authors report no conflicts of interest.
