# Supplementary material for: Opportunities for Individual- and Population-Specific Adaptations in Food Is Medicine: A Scoping Review
Source: Adv Nutr. 2026 Jun 18;17(8):100684. doi: 10.1016/j.advnut.2026.100684 (PMC13382255; doi:10.1016/j.advnut.2026.100684)
Supplement: multimedia component 1 [file mmc1.docx]

**Supplementary Table 1**. Detailed search strategy to identify relevant literature on adaptations used in Food is Medicine interventions

| **Search Criteria** | **Search Strategy** |
| --- | --- |
| ***Population:***  Not limited | “Healthy” OR “Health*” OR “Type 2 Diabetes Mellitus” OR “T2DM” OR “T2D*” OR “Chronic Disease” or “Chronic Condition” OR “Chronic*” OR “Medical*” OR “Healthcare” OR “Food Insecure” OR “Food Secure” OR “Hunger*” OR “Hungry” OR “Medical” OR “Medicaid” OR “Adults” OR “Children” OR “Adolesc*” OR “Teens” OR “youth” |
| ***Intervention:***  Food is Medicine | “Produce Prescription” OR “Medically Tailored*” OR “Medically Supported*” OR “Groceries” OR “Meals” OR “Food as Medicine” OR “Food is Medicine” |
| ***Condition:***  Age, disease state, or culture and community | “culturally modified” OR “cultur*” OR “modified” OR “adapted” OR “language” OR “bilingual” OR “race/ethnicity” OR “age” OR “disease” |
| ***Outcome:***  Not limited | “Fruit” OR “Vegetable” OR “Diet*” OR “HEI” OR “HbA1C” OR “Hemoglobin A1C” OR “Physical Activity” OR “Biomarkers” OR “Health*” OR “Primary*” OR “Second*” OR “Psycho*” OR “Wellness” OR “Well-being” OR “Social*” OR “mental*” OR “physical*” OR “obesity” OR “obes*” OR “BMI” OR “blood pressure” OR “hyperten*” |

**Location:**

United States

**Timeline:**

January 2000 – December 2024

**Article Types:**

Included: Randomized controlled trials; observational; cross-sectional

Excluded: Case reports; dissertations; reviews; systematic reviews; meta-analysis

**Language:**

English only
