# Supplementary material for: Opportunities for Individual- and Population-Specific Adaptations in Food Is Medicine: A Scoping Review
Source: Adv Nutr. 2026 Jun 18;17(8):100684. doi: 10.1016/j.advnut.2026.100684 (PMC13382255; doi:10.1016/j.advnut.2026.100684)
Supplement: multimedia component 2 [file mmc2.docx]

**Supplemental Table 2**. Characteristics of adaptation type included in this scoping review classified by FIM intervention type

|  | **MTM (N=20)** | **MTG (N=18)** | **PRx (N=51)** | **Overall (N=89)** |
| --- | --- | --- | --- | --- |
| **Adaptation Details** |  |  |  |  |
| Age & household size | 0 (0%) | 0 (0%) | 5 (9.8%) | 5 (5.6%) |
| Disease state | 6 (33.3%) | 16 (80.0%) | 5 (9.8%) | 27 (30.3%) |
| Culture & community | 2 (11.1%) | 1 (5.0%) | 17 (33.3%) | 20 (22.5%) |
| Age & household size; Culture & community | 2 (11.1%) | 0 (0%) | 4 (7.8%) | 6 (6.7%) |
| Age & household size; Disease state | 1 (5.6%) | 0 (0%) | 0 (0%) | 1 (1.1%) |
| Disease state; Culture & community | 3 (16.7%) | 2 (10.0%) | 5 (9.8%) | 10 (11.2%) |
| Age & household size; Disease state; Culture & community | 2 (11.1%) | 1 (5.0%) | 0 (0%) | 3 (3.4%) |
| Adaptations not detailed | 2 (11.1%) | 0 (0%) | 15 (21.6%) | 17 (19.1%) |

Abbreviations: MTG: Medically tailored groceries; MTM: Medically tailored meals; PRx: Produce Prescription Programs
